# Angiotensin Converting Enzyme 2 (ACE2) in Pregnancy: Preeclampsia and Small for Gestational Age

**DOI:** 10.3389/fphys.2020.590787

**Published:** 2020-09-30

**Authors:** Sonia Tamanna, Vicki L. Clifton, Kym Rae, Dirk F. van Helden, Eugenie R. Lumbers, Kirsty G. Pringle

**Affiliations:** ^1^Priority Research Centre for Reproductive Sciences, University of Newcastle, Newcastle, NSW, Australia; ^2^School of Biomedical Sciences and Pharmacy, Faculty of Health and Medicine, University of Newcastle, Newcastle, NSW, Australia; ^3^Pregnancy and Reproduction Program, Hunter Medical Research Institute, University of Newcastle, Newcastle, NSW, Australia; ^4^School of Medicine, Robinson Research Institute, University of Adelaide, Adelaide, SA, Australia; ^5^Mater Medical Research Institute and Translational Research Institute, University of Queensland, Brisbane, QLD, Australia

**Keywords:** angiotensin converting enzyme 2 (ACE2), angiotensin peptides, preeclampsia, pregnancy, small for gestational age

## Abstract

**Introduction:**

An imbalance in angiotensin (Ang) peptides could contribute to the pathophysiology of preeclampsia (PE) and poor fetal growth.

**Methods:**

We measured maternal plasma levels of Ang peptides and converting enzymes in non-pregnant women (*n* = 10), in normal pregnant women (*n* = 59), women delivering small for gestational age babies (SGA, *n* = 25) across gestation (13–36 weeks) and in women with PE (*n* = 14) in their third trimester.

**Results:**

Plasma ACE, ACE2, and Ang-(1-7) levels, and ACE2 activity were significantly higher in normal pregnant women compared with non-pregnant women; neprilysin (NEP) levels were not changed. In SGA pregnancies, ACE and ACE2 levels were higher in early-mid pregnancy compared with normal pregnant women. In women with PE, plasma ACE, ACE2, NEP, and Ang-(1-7) levels and ACE2 activity were lower than levels in normal pregnant women.

**Conclusion:**

The higher plasma ACE2 levels and activity in pregnancy could be driving the higher Ang-(1-7) levels. The early gestation increases in ACE and ACE2 levels in SGA pregnancies highlights the possibility that these enzymes could be used as potential early biomarkers of poor fetal growth. In women with PE, the reduced ACE2 and NEP levels at term, could be contributing to the reduction in Ang-(1-7) levels. These findings suggest that dysfunctional relationships between two key enzymes in the circulating RAS are involved in the pathogenesis of PE and SGA. Since soluble ACE2 can prevent binding of the novel coronavirus, SARS-CoV-2, to membrane bound ACE2, the interplay between ACE2 and the coronavirus and its impact in pregnancy requires further investigation.

## Introduction

During pregnancy, the renin-angiotensin system (RAS) plays a significant role in the regulation of blood pressure and salt and water balance. It is activated by estrogen-induced increases in angiotensinogen (AGT) and, subsequently, by the reduction in systemic vascular resistance caused by the ovarian hormone, relaxin (Lumbers and Pringle, 2014). It is perhaps not surprising then that alterations in the circulating and intrauterine RAS are associated with the development of preeclampsia (PE) and pregnancies in which the infant is born small for gestational age (SGA) (Irani and Xia, 2008; Zhou et al., 2013; Delforce et al., 2019).

**FIGURE 1:**
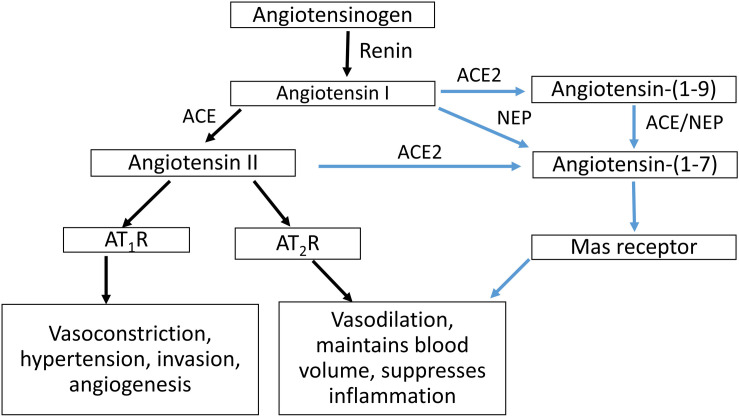
Schematic representation of the RAS cascade. ACE, angiotensin converting enzyme; ACE2, angiotensin converting enzyme 2; NEP, neprilysin; AT_1_R, Angiotensin II type 1 receptor, AT_2_R; Angiotensin II type 2 receptor.

The circulating RAS cascade (Figure 1) begins with the release of active renin from the kidney, which cleaves angiotensin I (Ang I) from AGT. Angiotensin II (Ang II), the major effector molecule of the RAS cascade, is cleaved from Ang I by angiotensin converting enzyme (ACE). Ang II elicits its effects by binding to the Ang II type 1 receptor (AT_1_R). Interactions between Ang II and the AT_1_R stimulate sodium retention (both directly and via stimulating the release of aldosterone) and raise blood pressure, both by central actions and direct effects on blood vessels. Alternatively, Ang II can bind to the AT_2_R, which has opposing actions than those of Ang II binding to the AT_1_R (Escobar et al., 2004).

The actions of Ang II/AT_1_R are counter-balanced by an alternate RAS axis (Figure 1). This axis encompasses ACE2, which can form Ang-(1-7) by removing a single amino acid from Ang II. In conjunction with ACE, ACE2 can also form Ang-(1–7) from Ang I, as can neprilysin (NEP) by acting on Ang I. Ang-(1-7) acting through the Mas receptor, opposes the actions of the Ang II/AT_1_R pathway, as do interactions between Ang II and the AT_2_R (Warner et al., 2004).

ACE2 plays a role in balancing the vasoconstrictor and vasodilator arms of the RAS (Gallagher et al., 2008) and exerts a protective role in end organ damage (heart, lung, kidney, etc.) (Oudit et al., 2009; Wang et al., 2016). ACE2 can undergo ectodomain shedding to secrete a soluble form of ACE2, sACE2 (Lambert et al., 2005).

ACE2 has an almost 500-fold higher catalytic efficiency in forming Ang-(1-7) from Ang II than in converting Ang I to Ang-(1-9) (Vickers et al., 2002), which is then cleaved by ACE. ACE2 is insensitive to ACE inhibitors such as captopril, lisinopril, and enalapril (Donoghue et al., 2000), and there is evidence that ACE2 counteracts the effects of the RAS mediated by over activity of Ang II and the AT_1_R (Chhabra et al., 2013).

Plasma Ang-(1-7) is increased in pregnancy, whereas ACE concentrations are decreased (Merrill et al., 2002). In women with PE, maternal plasma Ang-(1-7), Ang II and plasma renin activity are reduced compared with normotensive pregnant women (Merrill et al., 2002; Velloso et al., 2007). ACE concentrations are however, increased in women with PE (Merrill et al., 2002). Women who gave birth to SGA babies also had higher concentrations of plasma ACE at 15 weeks’ gestation compared with women who delivered babies of normal birth weight (Zhou et al., 2013). To the best of our knowledge, there are no data on changes in maternal ACE2 and NEP levels in either normal pregnant women or women suffering from complicated pregnancies. It is possible for Ang-(1-7) to be formed via the activity of both these pathways (see Figure 1).

ACE2 is the critical receptor for the highly pathogenic novel SARS-CoV-2 virus, which causes COVID-19. The SARS-CoV-2 spike protein binds with human ACE2 with substantially higher affinity than does the spike protein of SARS-CoV (Walls et al., 2020; Wan et al., 2020; Wrapp et al., 2020). Therefore, our particular interest in measuring sACE2 in pregnant women could be important in understanding the pathogenesis of SARS-CoV-2 in pregnancy.

In this study, we measured ACE, sACE2, Ang-(1-7) and NEP levels in plasma from women with uncomplicated (normal) pregnancies and compared them to levels found in healthy non-pregnant women. We also measured plasma levels of these components of the RAS in women with pregnancies complicated by either PE or SGA.

## Materials and Methods

### Study Population

Data and biobanked plasma samples from a cohort study undertaken at the Lyell McEwin Hospital, Adelaide, Australia, that was used to study the effects of asthma during pregnancy on the mother, placenta and baby, were examined (Dickinson et al., 2016). The study was approved by the Queen Elizabeth Hospital and Lyell McEwin Hospital Human Research Ethics Committee and the University of Adelaide Human Research Ethics Committee. Samples were collected from (*n* = 59) women with uncomplicated pregnancies at 13, 18, 30, and 36 weeks. Samples were also collected from 25 women with SGA pregnancies (13, 18, 30, and 36 weeks) and 5 women who developed PE (range: 30–36 weeks).

We used stored plasma samples from healthy non-pregnant women (*n* = 10), and women who developed preeclampsia (*n* = 9, 26–35 weeks of gestation, Supplementary Table 1) collected at the John Hunter Hospital and Tamworth Base Hospital, NSW, Australia. The collection and use of these samples were approved by the University of Newcastle Research Ethics Committee and Hunter New England Health Human Research Ethics Committee. The normal pregnancy group included individuals who delivered appropriately grown infants (>10^*th*^ centile for gestational age) at term (>37 weeks) who did not have hypertension, diabetes, infections, or renal dysfunction. Women were classified as having PE based on the SOMANZ definition (Lowe et al., 2015). However, some samples were collected prospectively, at which time we measured blood pressure. Pregnancies complicated by SGA had babies whose birth weights were <10^*th*^ centile for gestational age calculated using the GROW (Gestation Related Optimal Weight) method (https://www.gestation.net/).

### Blood Sampling

For all samples, maternal venous blood was collected in lithium/heparin tubes, centrifuged at 1000 g for 15 min, aliquoted and stored at −80°C until assays were performed.

### Measurement of Plasma ACE, ACE2, and NEP Levels

Maternal plasma ACE and NEP concentrations were determined using commercially available enzyme-linked immunosorbent assay kits according to the instructions of the manufacturer (Duoset, R&D Systems, Minneapolis, MN, United States). Concentrations of maternal plasma ACE2 were measured using commercial enzyme−linked immunosorbent assay (ELISA) kits (Cloud−Clone Corp, Houston, TX, United States). All samples were assayed in duplicate. The mean intra- and inter-assay coefficients of variations were 6.88% (*n* = 131) and 15.16% (5 plates) for ACE, 4.68% (*n* = 131) and 13.22% (6 plates) for ACE2, and 7.89% (*n* = 96) and 1.24% for NEP (4 plates), respectively.

### Determination of ACE2 Activity

The ACE2 activity assay was carried out according to a published protocol with minor modifications (Shao et al., 2013; Xiao and Burns, 2017). Briefly, 15 μL of each plasma sample was diluted with an enzyme buffer consisting of 1 M NaCl, 75 mM Tris-HCl, 5.0 mM ZnCl_2_, pH 6.5 with protease inhibitors, which were 10 μM captopril, 5 μM amastatin, and 10 μM bestatin (all from Sigma Aldrich, St. Louis, Missouri, United States) and 10 μM Z-prolyl-prolinal (Enzo Life Sciences, Inc., NY, United States). The ACE2-specific quenched fluorescent substrate, MCA-Ala-Pro-Lys-2, 4 dinitrophenyl (AnaSpec, Inc., San Diego, CA, United States) diluted in enzyme buffer were added to the samples in a final concentration of 50 μM in 100 μL on a black 96-well microplate. The plate was covered with aluminum foil to protect from light and incubated at room temperature for 24 h on a plate shaker. We have used recombinant human ACE2 (R&D Systems, Minneapolis MN) as the standard instead of using a fluorescent product to measure ACE2 activity. ACE2 cleaves the Pro-Lys bond of the substrate, and the Relative Fluorescence Units (RFU) of the released Mca-Ala-Pro was measured over a period of 24 h. The plate was read on a fluorescence reader (Clario Star, BMG Labtech, GmbH, Ortenberg, Germany) with an excitation wavelength of 320 nm and emission wavelength of 405 nm. The specificity of the enzyme activity was checked using 1 μM of an ACE2 inhibitor (DX600; AnaSpec, Inc., San Diego, CA, United States), which is specific for human ACE2. We calculated how much ACE2 activity there was in a sample comparing the RFU to the RFU of known concentrations of recombinant ACE2, that were treated in an identical manner. Thus ACE2 activity is reported as the activity/ng/ml of ACE2. All samples were assayed in duplicate. The mean intra and inter-assay coefficients of variations were 7.17% (*n* = 136) and 4.16% (5 plates), respectively.

### Measurement of Angiotensin-(1-7)

A direct radioimmunoassay (RIA) was used to measure plasma Ang-(1-7) levels by Prosearch Pty Ltd (Malvern, VIC, Australia) as described previously (Sykes et al., 2014b).

### Statistical Analyses

Data for all RAS enzyme levels and activity, as well as Ang-(1-7) levels, ACE2/ACE ratio, and ACE2 activity/ACE2 ratio, were expressed as median and interquartile ranges. A non-parametric Kruskal–Wallis test (with Dunn’s multiple comparison test) was performed to compare ACE, ACE2, NEP and Ang-(1-7) levels and ACE2 activity, ACE2/ACE ratio, and ACE2 activity/ACE2 ratio between non-pregnant women and women with normal pregnancies (at 13, 18, 30, and 36 weeks of gestation). Mixed effect analysis (with Fisher’s LSD multiple comparison test) was used to compare differences between normal pregnancies and pregnancies complicated by SGA at each gestational age. A Mann–Whitney test was used to determine differences between normal pregnancies and women with PE in the third trimester. Spearman’s correlations were used to determine any association between ACE2 and Ang-(1-7) levels and ACE2 levels as well as birth weight centile. Differences in demographic characteristics of different pregnant groups were identified by the chi-square test. Differences were considered significant if *P* < 0.05.

## Results

### Baseline Characteristics of the Study Population

A total of 10 non-pregnant women were recruited in this study. The median age was 26.5 years (interquartile range (IQR): 25–35 years) and BMI was 25.32 kg/m^2^ (IQR: 22.83–28.30). Three of the ten non-pregnant women were taking oral contraceptives, and a further four were using hormonal contraceptive devices (e.g., Implanon and Mirena).

Table 1 outlines the baseline characteristics of each pregnancy group. Maternal age, BMI and ethnicity were not significantly different between the study groups. The median birth weights and birth weight centiles of SGA infants were less (*P* < 0.001 and *P* < 0.001, respectively), as were the birth weights and birth weight centiles of infants whose mothers had PE (*P* < 0.001 and *P* = 0.002, respectively) than those from normal pregnancies. Women with PE delivered at an earlier gestational age compared with those who had normal pregnancies (P < 0.001), which could account for their lower birth weights. No significant difference was observed in the gestational age at delivery of SGA babies compared with those born from women with normal pregnancies. The number of smokers was significantly higher in women delivering SGA babies compared to those with normal pregnancy outcomes (*P* = 0.028), whereas there was no significant differences between the prevalence of smokers between the PE and normal pregnancy groups. The number of male babies was higher (*P* = 0.002) in the PE group compared to normal pregnant group. Two of the women in the PE group were classified as having early-onset PE (developing < 34 weeks gestation) and seven of the women delivered a baby who was SGA.

**TABLE 1:** Baseline characteristics of normal, PE and SGA pregnancies.

	**Normal Pregnancy *n* = 59**	**PE *n* = 14**	**SGA *n* = 25**
Maternal age (years)	26 (23–30.3)	26.6 (22.8–29.0)	27.5 (19–31)
BMI (kg/m^2^)	26.6 (23.1–30.4)	27.97 (24.9–32.4)	29.8 (25.7–34.2)
GA at delivery (weeks)	39.6 (38.4–40.3)	35.53 (33.8–38.0)*	39.7 (38.2–40.9)
Birth weight (g)	3460 (3140–3735)	2395 (1553–2908)*	2830 (2543–3080)*
BWC	46.40 (27.6–75.0)	9.2 (0.4-56.4)*	5.25 (1.8–7.2)*
Maternal ethnicity			
Caucasian	43 (72.88)	12 (85.71)	20 (80)
Not Caucasian	16 (27.12)	2 (14.29)	5 (20)
Smoker	14 (23.73)	02 (14.29)	12 (48)*
Infant sex			
Male	28 (47.46)	13 (92.86)*	10 (40)
Female	31 (52.54)	1 (7.14)	15 (60)
13 weeks			
sBP (mmHg)	110 (105–115)	−	111 (109–125)
dBP (mmHg)	70 (60–70)	−	70 (60–75)
18 weeks			
sBP (mmHg)	109 (100–120)	−	112 (110–121)
dBP (mmHg)	64 (60–70)	−	70 (70–80)
30 weeks			
sBP (mmHg)	112 (102–120)	−	117 (106–123)
dBP (mmHg)	70 (60–70)	−	70 (60–80)
36 weeks			
sBP (mmHg)	110 (100–120)	−	110 (100–129)
dBP (mmHg)	70 (64–80)	−	72 (62–80)
Third trimester (26–36 weeks)			
sBP (mmHg)	110 (100–120)	120 (110.5–136)	−
dBP (mmHg)	70 (64–80)	80 (70–83)	−

Data are expressed as median (IQR) or number (%) for maternal ethnicity, smoker, and infant sex. BMI, Body Mass Index; BWC, birth weight centile; dBP, diastolic blood pressure; GA, gestational age; PE, preeclampsia; sBP, systolic blood pressure; SGA, small for gestational age. *indicates a significant difference compared with the normal pregnancy group.

There were no significant differences in the systolic or diastolic blood pressures of women with normal pregnancies and those with SGA at any gestational age (13, 18, 30, or 36 weeks, Table 1). There were no differences in systolic and diastolic blood pressures in the PE group compared with the normal (normotensive) pregnancy group at the time of sampling in the third trimester (Table 1).

### Associations Between ACE2 Activity and ACE2 Levels as Well as Between ACE2 and Ang-(1-7) in Non-pregnant and Pregnant Women

**FIGURE 2:**
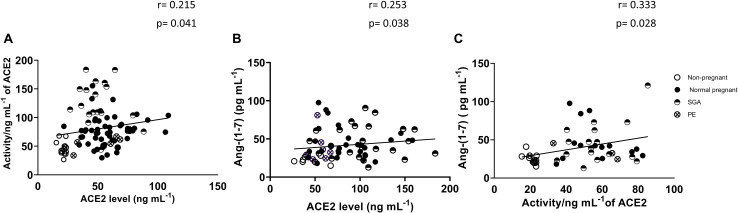
Correlations between **(A)** ACE2 levels and ACE2 activity (non-pregnant and all pregnancy groups combined), **(B)** ACE2 levels and Ang-(1-7) levels, and **(C)** ACE2 activity and Ang-(1-7) levels.

Figure 2 shows the association between plasma ACE2 activity and ACE2 levels as well as the association between plasma ACE2 and Ang-(1-7) in non-pregnant and pregnant women (normal, PE and SGA). ACE2 activity was positively correlated with ACE2 levels (*r* = 0.215, *P* = 0.041). Plasma Ang-(1-7) levels were positively correlated with ACE2 levels and activity (*r* = 0.253, *P* = 0.038 and *r* = 0.333, *P* = 0.028, respectively).

### ACE, ACE2, NEP, and Ang-(1-7) Levels, ACE2 Activity and ACE2/ACE, ACE2 Activity/ACE2 Ratio in Normal Pregnancies

**FIGURE 3:**
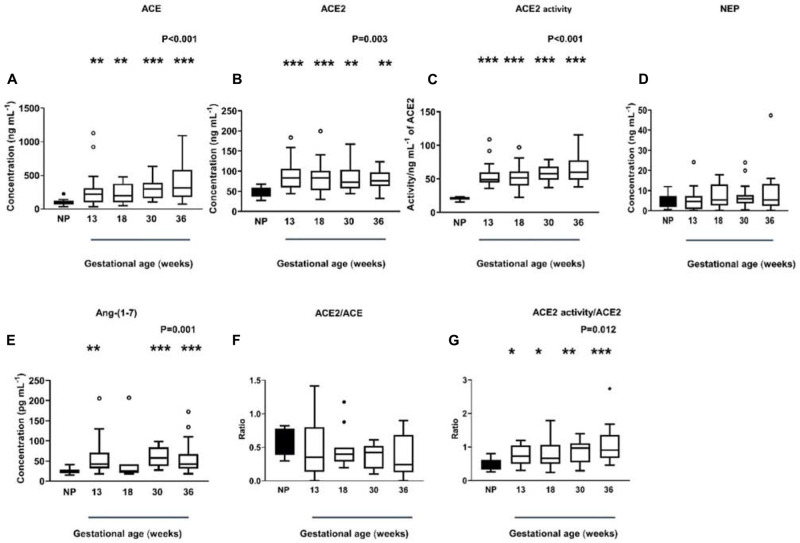
Plasma levels and activity of ACE, ACE2, and NEP in non-pregnant (NP) and pregnant women **(A–D)**. Plasma levels of Ang-(1-7) were measured by radioimmunoassay in NP and pregnant women **(E)**. Plasma ACE2/ACE ratio and ACE2 activity/ACE ratio in NP and pregnant women **(F,G)**. Data are expressed as median and interquartile range. *n* = 9–10 samples for the NP group (black box), *n* = 7–35 samples/group for 13, 18, 30, and 36 weeks of normal pregnancy (white box). *P*-values were calculated using a Kruskal–Wallis test (with Dunn’s multiple comparison test). *P < 0.05, **P < 0.01, ***P < 0.001 versus NP.

Plasma ACE levels were significantly higher in women with normal pregnancies compared with non-pregnant women (*P* < 0.001) and remained high throughout gestation (Figure 3A). Women with normal pregnancies also had elevated ACE2 levels (*P* = 0.003) and activity (*P* < 0.001) compared with non-pregnant women (Figures 3B,C). However, ACE2 levels and activity did not change across gestation. NEP levels in non-pregnant and pregnant women were similar and did not change across gestation (*P* = 0.492; Figure 3D).

In women with normal pregnancies, we determined if the known rise in plasma Ang II in pregnancy was counter-balanced by elevated Ang-(1-7) levels (Figure 3E). Plasma Ang-(1-7) levels were significantly increased in normal pregnant women compared with non-pregnant women (*P* = 0.001, Figure 3E), this was statistically significant at all gestational ages examined except at 18 weeks of gestation.

We also examined the ACE2/ACE ratio (Figure 3F) as well as the ratio between ACE2 activity/ACE2 levels (Figure 3G) in normal pregnant women. In pregnancy, the ACE2/ACE ratio did not change across gestation (*P* = 0.243). However, the ACE2 activity/ACE2 ratio was significantly higher in normal pregnant women (*P* = 0.012) and remained elevated throughout gestation (Figure 3G).

### ACE, ACE2, NEP, and Ang-(1-7) Levels, ACE2 Activity and the ACE2/ACE and ACE2 Activity/ACE2 Ratios in SGA Pregnancies

**FIGURE 4:**
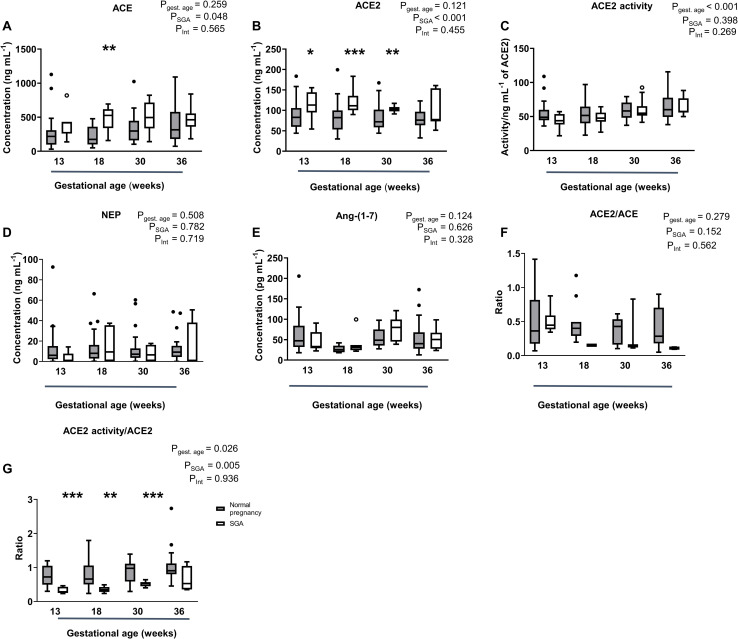
Plasma levels and activity of **(A)** ACE, **(B,C)** ACE_2_, and **(D)** NEP as well as levels of **(E)** Ang-(1-7), **(F)** ACE2/ACE ratio, and **(G)** ACE2 activity/ACE2 ratio in normal and SGA pregnancies. Data are expressed as median and interquartile range. *n* = 5–36 samples/group for normal pregnancies (gray box) and *n* = 4–15 samples/group for SGA pregnancies (white box). *P*-values were calculated using a two-way ANOVA (mixed effect model with Fisher’s LSD multiple comparison test). ^∗^*P* < 0.05, ^∗∗^*P* < 0.01, ^∗∗∗^*P* < 0.001 versus normal pregnancy group of the same gestational age.

Women delivering babies who were born SGA had levels of ACE and ACE2 that were significantly higher than those in women with normal pregnancies (*P* = 0.048 and *P* < 0.001; Figures 4A,B). When examining the differences at each gestational age, the only statistical differences were at 18 weeks for ACE levels and at 13, 18, and 30 weeks for ACE2 levels. ACE2 activity however was not significantly different between normal and SGA pregnancies (Figure 4C). NEP and Ang-(1-7) levels in SGA pregnancies were similar to those found in women with normal pregnancies, as was the ACE2/ACE ratio (Figures 4D–F). However, the ratio between ACE2 activity and ACE2 levels was significantly decreased in women with SGA pregnancies compared with normal pregnancies (*P* = 0.005; Figure 4G), this was significant at 13, 18, and 30 weeks of gestation but not at 36 weeks.

## ACE, ACE2, NEP, and Ang-(1-7) Levels, ACE2 Activity, and the ACE2/ACE Ratio in Women With Preeclampsia

**FIGURE 5:**
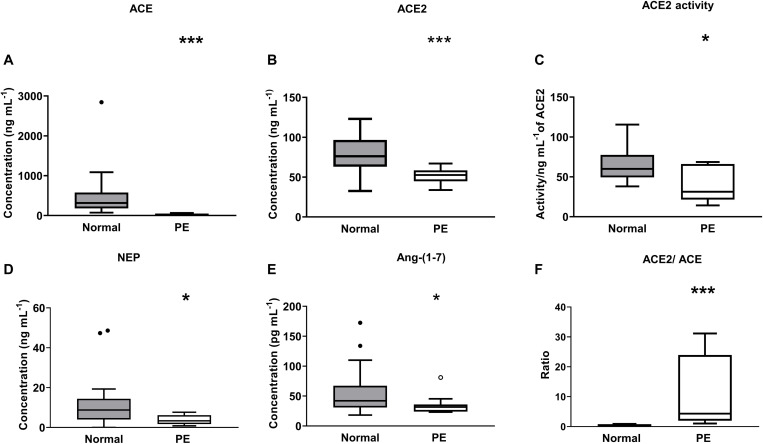
Plasma levels and activity of **(A)** ACE, **(B,C)** ACE2, and **(D)** NEP as well as **(E)** levels of Ang-(1-7), and **(F)** ACE2/ACE ratio in the third trimester (≥26 weeks of gestation) in women with normal pregnancies (gray box) and women with preeclampsia (PE; white box). Data are expressed as median and interquartile range. *n* = 16–32 for normal pregnancy group and *n* = 8–14 samples for the PE group. *P < 0.05, **P < 0.01, ***P < 0.001 versus normal pregnancy group.

ACE levels were significantly decreased in women with preeclampsia compared with levels measured in women with normal pregnancies (*P* < 0.001; Figure 5A). Both plasma ACE2 levels (*P* < 0.001) and activity (*P* = 0.024) were significantly lower in women with preeclampsia compared with women with normal pregnancies (Figures 5B,C). There was no significant difference between the PE alone and PE with SGA samples in terms of ACE2 levels (data not shown). Plasma NEP levels were also decreased in women with PE (*P* = 0.024; Figure 5D).

Figure 5E shows the plasma Ang-(1-7) levels in women with normal pregnancies and women with PE. Women with PE had reduced levels of plasma Ang-(1-7) compared with levels in women with normal pregnancies (*P* = 0.034; Figure 5E). The ACE2/ACE ratio was increased in PE compared with normal pregnancies (*P* < 0.001; Figure 5F).

### Associations Between ACE, ACE2, ACE2 Activity and Birth Weight Centile

Table 2 shows the associations between ACE, ACE2 levels, and ACE2 activity and birth weight centile (all pregnant groups). Significant negative correlations were found between both ACE and ACE2 levels and birth weight centile (ACE: *r* = −0.488, *P* = 0.010, *r* = −0.384, *P* = 0.044 at 18 weeks and 30 weeks of gestation, ACE2: *r* = −0.289, *P* = 0.044, *r* = −0.627, *P* < 0.001, *r* = −0.405, *P* = 0.007 for 13, 18, and 30 weeks of gestation; Table 2). ACE2 activity was not associated with birth weight centile.

**TABLE 2:** Correlations between maternal sACE, sACE2, and sACE2 activity and birth weight centile (BWC).

	13 weeks	18 weeks	30 weeks	36 weeks
sACE vs. BWC	*r* = −0.294 *P* = 0.129	*r* = −0.488 *P* = 0.010*	*r* = −0.338 *P* = 0.044*	*r* = −0.262 *P* = 0.170
sACE2 vs. BWC	*r* = −0.289 *P* = 0.044*	*r* = −0.627 *P* < 0.001*	*r* = −0.405 *P* = 0.007*	*r* = −0.013 *P* = 0.936
sACE2 activity vs. BWC	*r* = 0.361 *P* = 0.054	*r* = 0.050 *P* = 0.789	*r* = −0.041 *P* = 0.826	*r* = −0.175 *P* = 0.404

Data show Spearman’s correlation coefficient. *indicates significant correlations.

## Discussion

In this study, we showed for the first time that ACE and ACE2 levels and activity were increased in the maternal circulation during pregnancy and remained high throughout gestation, however NEP levels were unchanged. Plasma ACE2 levels were approximately 68% to 71% higher across gestation in women with normal pregnancies compared with non-pregnant women. We also confirmed that Ang-(1-7) levels were increased in pregnancy compared with non-pregnant women. In addition, we found that ACE2 levels were positively associated with ACE2 activity and that Ang-(1-7) levels were positively associated with both ACE2 levels and activity. This latter observation suggests that the amount and activity of ACE2 in the plasma is rate-limiting in terms of the production of Ang-(1-7). Whether or not this also applies to membrane bound ACE2 needs to be determined. The increased activity of ACE2 in normal pregnancy suggests that it may play a role, through the production of Ang-(1-7), in the regulation of maternal blood pressure. Previous studies carried out in ACE2 knockout mice show that ACE2 deficiency is associated with an increase in maternal blood pressure during pregnancy (Yamaleyeva et al., 2015). Several studies have also shown that polymorphisms in the ACE2 gene are associated with an increased risk of hypertension (Malard et al., 2013; Patnaik et al., 2014).

In women who gave birth to SGA neonates, circulating ACE levels were increased at 18 weeks of gestation compared with those found in women with normal pregnancies. Elevated levels of maternal plasma ACE have also been documented by Zhou et al. in women at 15 weeks’ gestation who went on to deliver SGA neonates (Zhou et al., 2013). We found increased ACE2 levels in SGA pregnancies at 13, 18, and 30 weeks of gestation, but not in late pregnancy (36 weeks). It is possible, since ACE levels were increased at 18 weeks of gestation, that there was an elevation in ACE2 levels in early-mid gestation in order to regulate the activity of the Ang II via the AT_1_R pathway. Since ACE levels were not elevated at term, there was a corresponding reduction in ACE2 levels. ACE2 activity was not significantly different across gestation in women who later delivered SGA neonates compared with women with normal pregnancies. The increase in ACE2 levels in the absence of any change in ACE2 activity could be explained by the presence of an endogenous inhibitor, which has been described in human plasma (Lew et al., 2008). Further studies are obviously required to investigate if this inhibitor is elevated in SGA pregnancies compared with normal pregnancies.

Interestingly, a negative correlation was found in early pregnancy between birth weight centiles and ACE and ACE2 levels (Table 2) suggesting that the increased production of angiotensin enzymes in early gestation could influence birth weight. However, in late gestation, any such effect was no longer apparent. We also acknowledge that BMI and parity might influence the levels and activity of RAS enzymes as well as Ang-(1-7). There were however no significant correlations between BMI and ACE, ACE2 levels, ACE2 activity, and Ang-(1-7) (data not shown).

This is the first study to measure plasma ACE2 in SGA pregnancies. ACE2 mRNA is present in almost all human tissues in particular in the kidney, heart, lung, testis and gastrointestinal tissues, where it has critical regulatory functions (Harmer et al., 2002). Indeed, ACE2 metabolizes Ang II and reduces its levels and produces the vasodilator peptide, Ang-(1-7). Therefore, it is critical for regulating the actions of Ang II mediated by its AT_1_R. A previous study from our group showed that placental ACE2 mRNA expression was lower in pregnancies associated with fetal growth restriction (Delforce et al., 2019), suggesting that reduced placental ACE2 levels and reduced Ang-(1-7) production might contribute to the etiology of SGA. The elevation in plasma ACE2 levels in SGA, taken together with the reduction in placental ACE2, suggests, however, that placental ACE2 is not contributing to the high levels of sACE2 in the maternal circulation in SGA pregnancies. Other tissues that could contribute ACE2 to the maternal circulation include the kidney, which has high levels of ACE2 and a 50% increase in renal blood flow during pregnancy (Brosnihan et al., 2003).

Further studies are required to investigate the relationships between placental ACE and ACE2 mRNA expression and activity and plasma ACE and ACE2 levels and activity in matched samples from SGA pregnancies to fully elucidate the effects of these two enzymes in the etiology of SGA. These studies could also be used to determine if ACE and ACE2 are early biomarkers for SGA or fetal growth restriction (see Zhou et al., 2013).

Samples from women with PE were collected from two different locations in Australia (New South Wales and South Australia). Although the methods of sample collection were very similar at the 2 sites we acknowledge that it could have influenced the levels of ACE2, ACE, NEP and Ang-(1-7). However, the clinical characteristics of these PE subgroups were similar (Supplementary Table 1) and there were no significant differences in levels of RAS enzymes and Ang peptides in samples collected from these two sites (Supplementary Figure 1). We also showed that there were no significant changes in levels/activity of RAS enzymes and Ang-(1-7) across gestation in women with PE (Supplementary Figure 2). Another limitation of the study is that in some cases samples were collected before the onset of PE and, in other cases, women may have been taking anti- hypertensive medications to control blood pressure. Both of these factors could influence the levels and activity of RAS components. Unfortunately, we do not have access to BP measurements from women after PE was diagnosed nor information on medications taken by the women. Therefore, some degree of caution should be taken in interpreting our findings.

Women with PE were found to have lower ACE and ACE2 (levels and activity) than women with normal pregnancies. This decrease in the angiotensin processing enzymes would be expected to result in altered Ang peptide levels in women with PE. We found that Ang-(1-7) levels were reduced in women with PE compared with women with normal pregnancies, and it is well known that levels of Ang II are suppressed in PE (Merrill et al., 2002; Velloso et al., 2007; Brosnihan et al., 2020). The decrease in Ang II could be due to the reduction in ACE and the increased amount of ACE2 relative to ACE (Figure 5F). In addition, we have shown for the first time that there is decreased levels of NEP in women with PE. Decreased levels of both ACE2 as well as NEP could account for the decreased levels of Ang-(1-7) in women with PE.

Importantly, some of the women in the PE group delivered SGA babies. In the SGA group, ACE2 was elevated in early gestation but in the PE group, maternal circulating ACE2 levels and activity were suppressed at term. This could be due to the timing of sampling (early gestation vs. term), since we have previously shown that Ang-(1-7) levels are similarly increased at 15 weeks of gestation in women who go on to develop preeclampsia (Sykes et al., 2014a), whereas at term Ang-(1-7) levels are reduced.

ACE2 protects tissues from the pro-inflammatory effects of Ang II by metabolizing it and producing Ang-(1-7) (Jia, 2016). As well, ACE2 is the receptor for SARS-CoV-2. Ang II has pro-inflammatory effects similar to those caused by the SARS virus. In acute respiratory distress syndrome recombinant human ACE2 reduces SARS-induced lung injury (Imai et al., 2005) and has been shown to prevent cell infection by SARS-Cov-2 (Monteil et al., 2020; Tai et al., 2020). Therefore, the high levels of sACE2 seen in normal pregnant women might protect the lungs from SARS-CoV-2 induced lung injury and slow down viral entry into cells thus reducing viral spread (Turner et al., 2004; Zhang et al., 2020). Conversely, the low levels of sACE2 found in pregnant women with PE might increase their susceptibility to COVID-19.

## Conclusion

In conclusion, our findings on the relationships between ACE and ACE2 in normal pregnancy and pregnancies associated with PE or SGA show that dysfunctional relationships between these two key enzymes in the circulating RAS could be involved in the pathogenesis of PE and SGA. Furthermore, increased levels of sACE2 in pregnant women could be significant in understanding the clinical effects of COVID-19.

## Data Availability Statement

The raw data supporting the conclusions of this article will be made available by the authors, without undue reservation.

## Ethics Statement

The studies involving human participants were reviewed and approved by the Queen Elizabeth Hospital and Lyell McEwin Hospital Human Research Ethics Committee, the University of Adelaide Human Research Ethics Committee, the University of Newcastle Research Ethics Committee, and the Hunter New England Health Human Research Ethics Committee. The patients/participants provided their written informed consent to participate in this study.

## Author Contributions

ST substantially contributed to the design of the study, acquisition of data, analysis and interpretation of data, revision of the draft article for content, and the final approval of the version submitted for publication. VC and KR provided plasma samples and contributed to the revision of the draft article for content and final approval of the version submitted for publication. DH contributed to the interpretation of the data, revision of the drafted article, and the final approval of the version to be published. EL substantially contributed to the interpretation of data, article drafting and revision for important intellectual content, and the final approval of the version to be published. KP substantially contributed to the design of the study, analysis and interpretation of data, revision of the draft article for content, and the final approval of the version submitted for publication. All authors contributed to the article and approved the submitted version.

## Conflict of Interest

The authors declare that the research was conducted in the absence of any commercial or financial relationships that could be construed as a potential conflict of interest.
