# Corrigendum: Angiotensin Converting Enzyme 2 (ACE2) in Pregnancy: Preeclampsia and Small for Gestational Age

**DOI:** 10.3389/fphys.2021.692761

**Published:** 2021-05-20

**Authors:** Sonia Tamanna, Vicki L. Clifton, Kym Rae, Dirk F. van Helden, Eugenie R. Lumbers, Kirsty G. Pringle

**Affiliations:** ^1^Priority Research Centre for Reproductive Sciences, University of Newcastle, Newcastle, NSW, Australia; ^2^School of Biomedical Sciences and Pharmacy, Faculty of Health and Medicine, University of Newcastle, Newcastle, NSW, Australia; ^3^Pregnancy and Reproduction Program, Hunter Medical Research Institute, University of Newcastle, Newcastle, NSW, Australia; ^4^School of Medicine, Robinson Research Institute, University of Adelaide, Adelaide, SA, Australia; ^5^Mater Medical Research Institute and Translational Research Institute, University of Queensland, Brisbane, QLD, Australia

**Keywords:** angiotensin converting enzyme 2 (ACE2), angiotensin peptides, preeclampsia, pregnancy, small for gestational age

In the original article, there was a mistake in Figure 3A and **G** as published. In Figure 3A it was written P=0<.001 instead of P<0.001 and Figure 3G it was written P=0.0012 instead of P=0.012. The corrected Figure 3 appears below.

**Figure 3:**
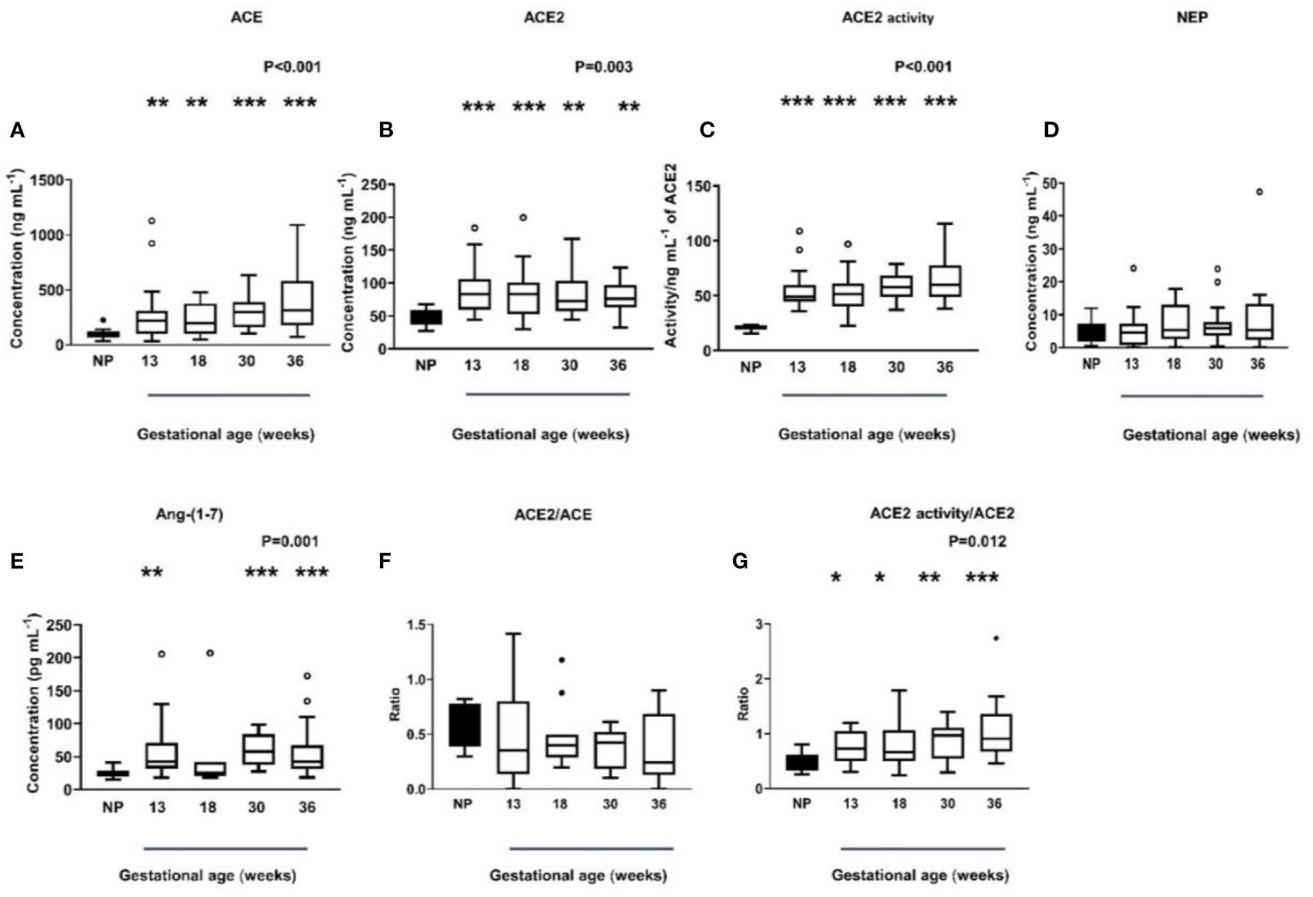
Plasma levels and activity of ACE, ACE2, and NEP in non-pregnant (NP) and pregnant women **(A–D)**. Plasma levels of Ang-(1-7) were measured by radioimmunoassay in NP and pregnant women **(E)**. Plasma ACE2/ACE ratio and ACE2 activity/ACE ratio in NP and pregnant women **(F,G)**. Data are expressed as median and interquartile range. *n* = 9–10 samples for the NP group (black box), *n* = 7–35 samples/group for 13, 18, 30, and 36 weeks of normal pregnancy (white box). *P*-values were calculated using a Kruskal–Wallis test (with Dunn's multiple comparison test). *P < 0.05, **P < 0.01, ***P < 0.001 versus NP.

In the original article, there was an error. In the results section, one of the *P*-values was stated incorrectly.

A correction has been made to Results, ACE, ACE2, NEP, and ANG-(1-7) levels, ACE2 activity, and the ACE2/ACE ratio in women with preeclampsia, paragraph 2:

“**Figure 5E** shows the plasma Ang-(1-7) levels in women with normal pregnancies and women with PE. Women with PE had reduced levels of plasma Ang-(1-7) compared with levels in women with normal pregnancies (*P* = 0.034; **Figure 5E**). The ACE2/ACE ratio was increased in PE compared with normal pregnancies (*P* < 0.001; **Figure 5F**).”

The authors apologize for this error and state that this does not change the scientific conclusions of the article in any way. The original article has been updated.

