# Supplementary material for: Angiotensin Converting Enzyme 2 (ACE2) in Pregnancy: Preeclampsia and Small for Gestational Age
Source: Front Physiol. 2020 Sep 30;11:590787. doi: 10.3389/fphys.2020.590787 (PMC7554608; doi:10.3389/fphys.2020.590787)
Supplement: Supplementary file 1 [file Data_Sheet_1.PDF]

Supplementary Table 1: Clinical characteristics of PE group based on locations

	**NSW (n=9)**	**SA (n= 5)**
Maternal age (years)	26.0 (21.6-28.5)	27.0 (22.5-31.5)
BMI (kg m^{-2})	26.3 (23.5-32.0)	32.1 (26.7-36.0)
GA at delivery (weeks)	35.2 (32.3-36.9)	36.0 (33.6- 38.3)
GA at sampling (weeks)	31.2 (28.0-33.3)	30.0 (30.0-33.0)
Birth weight (g)	2395 (1503-2795)	2850 (1743-3220)
BWC	14.1 (0.8-56.3)	9.2 (2.8-62.0)
SGA	4	3
**Third trimester (26-36 weeks)**		
sBP (mmHg)	129 (117-144)	111 (99-120)
dBP (mmHg)	79 (66-81)	86 (73-90)

Data are expressed as median (IQR) or number. BMI: body mass index; dBP: diastolic blood pressure; GA: gestational age; PE: preeclampsia; sBP: systolic blood pressure; BWC: birth weight centile; NSW: New South Wales; SA: South Australian; No differences were found between PE groups according to the site of collection.
