# Supplementary figures and images for: Angiotensin Converting Enzyme 2 (ACE2) in Pregnancy: Preeclampsia and Small for Gestational Age
Source: Front Physiol. 2020 Sep 30;11:590787. doi: 10.3389/fphys.2020.590787 (PMC7554608; doi:10.3389/fphys.2020.590787)

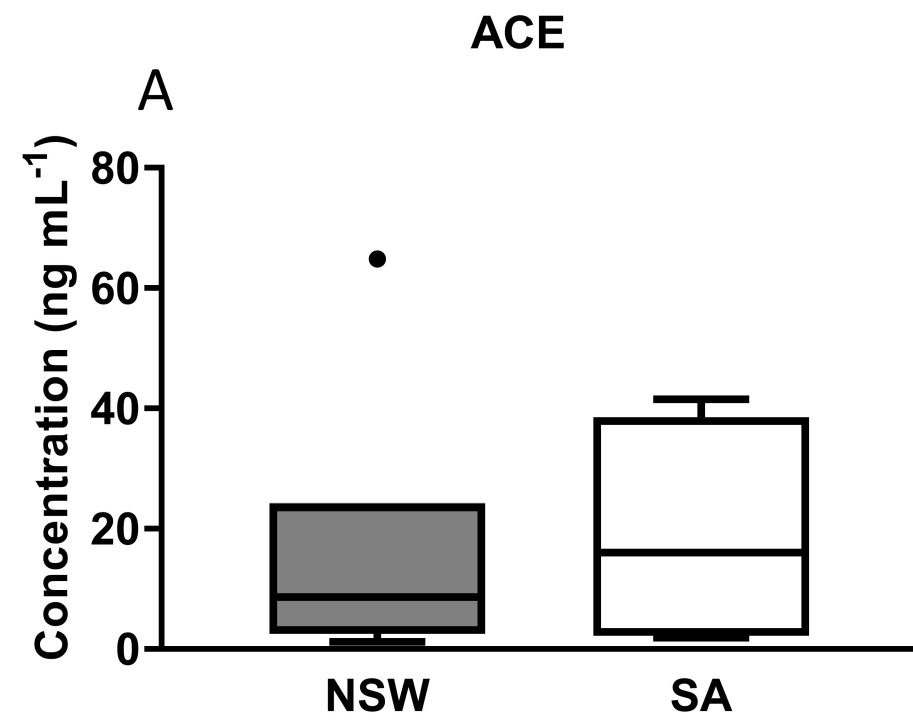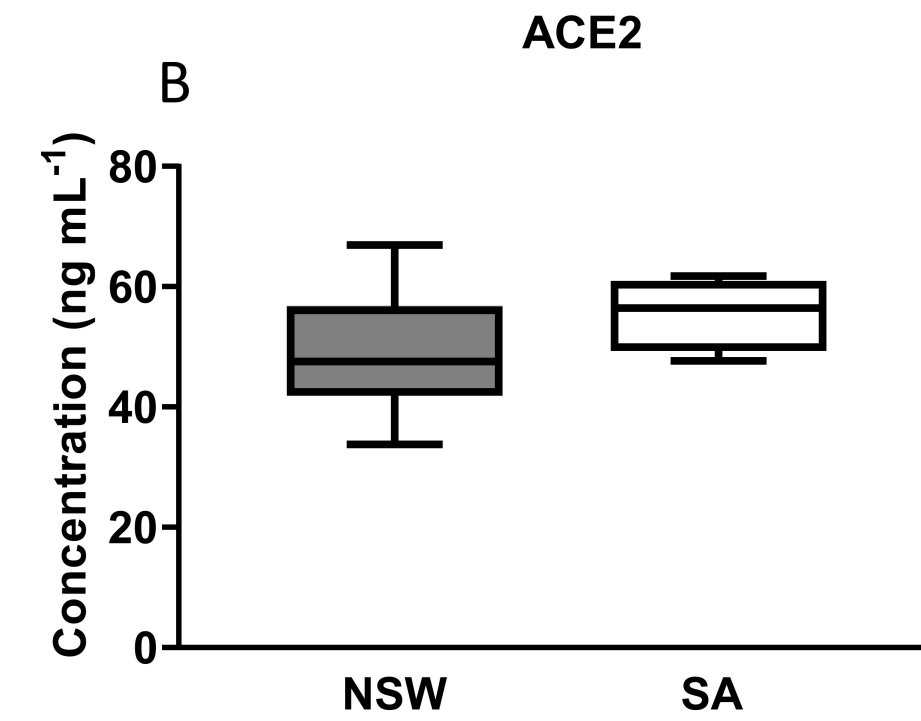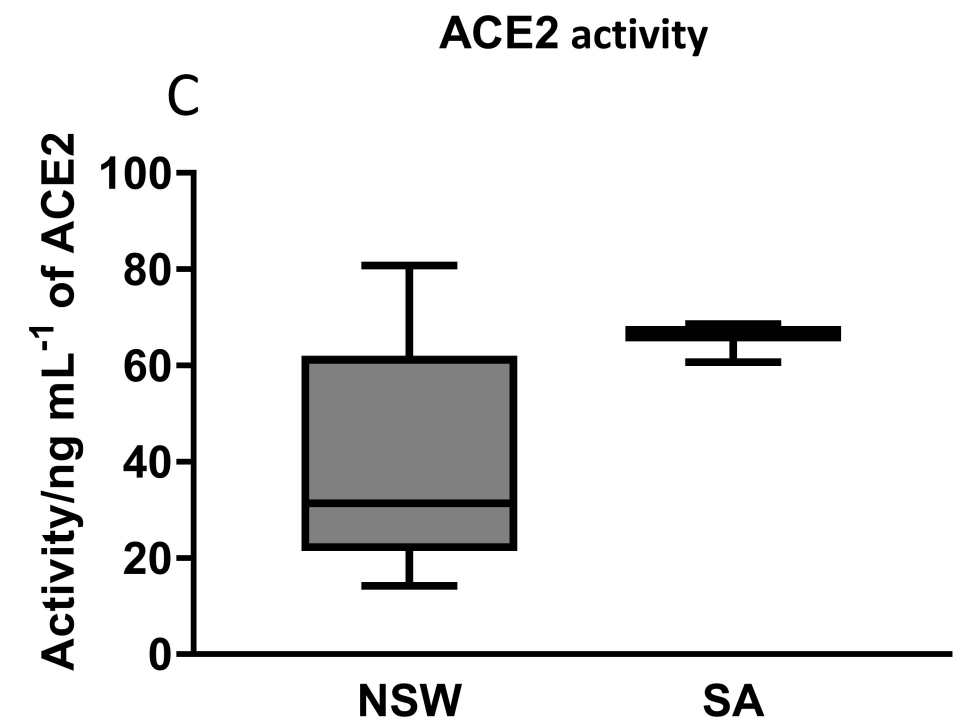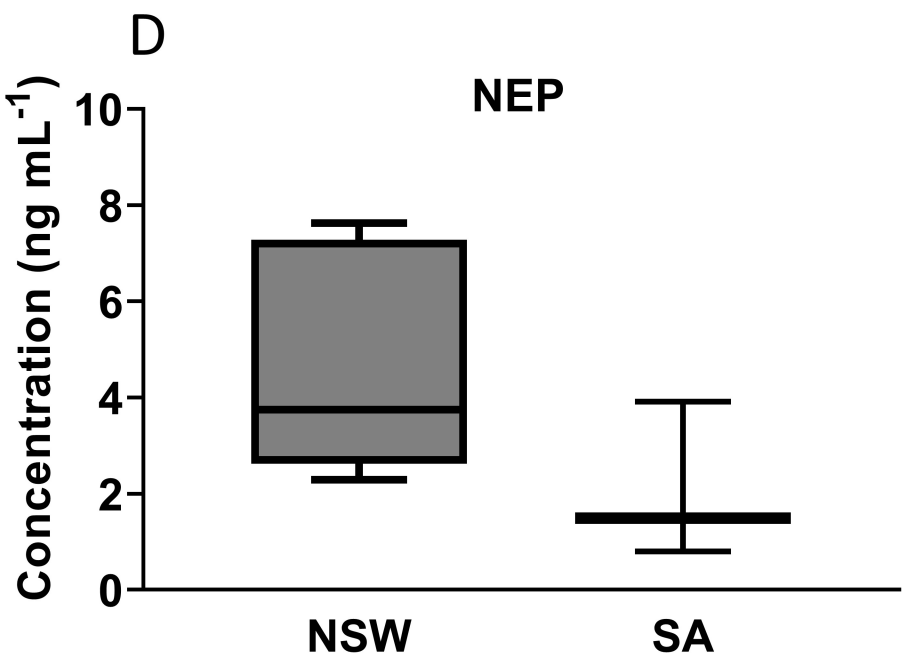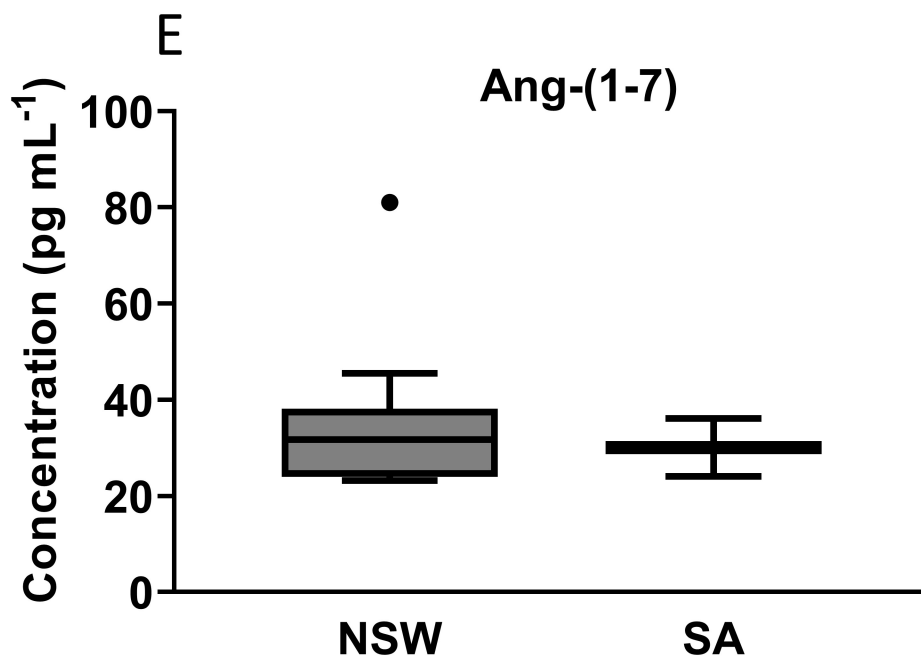

Supplement: Supplementary file 2 [file Data_Sheet_2.PDF]

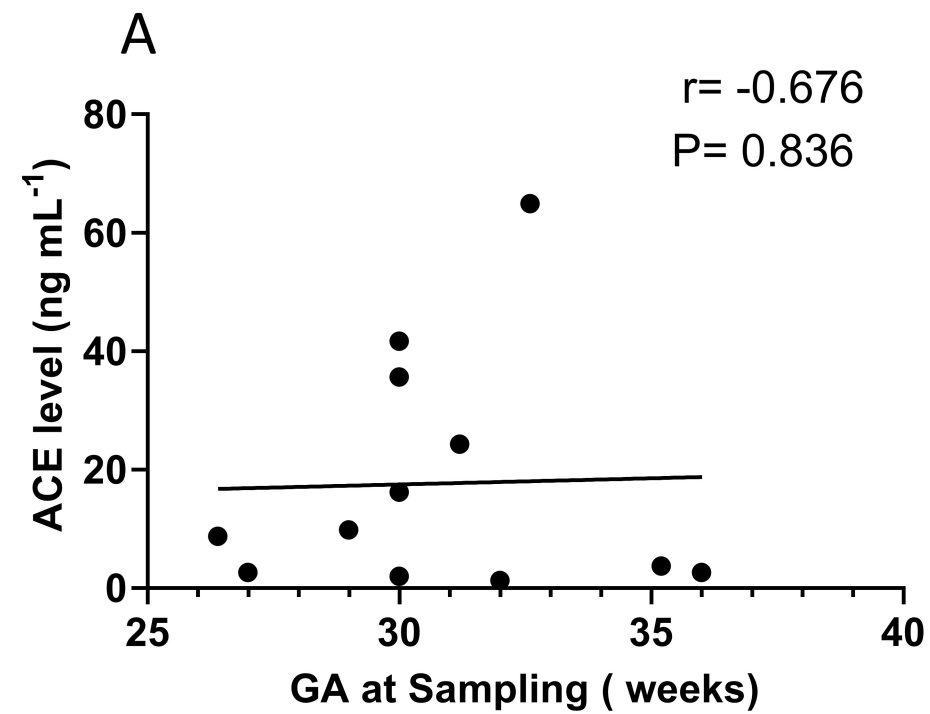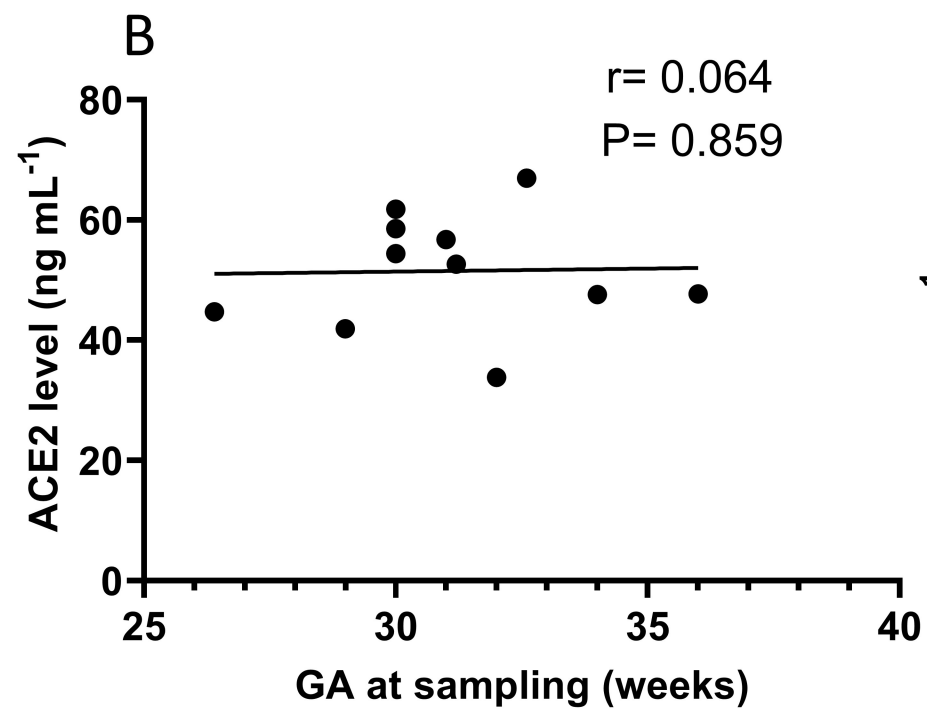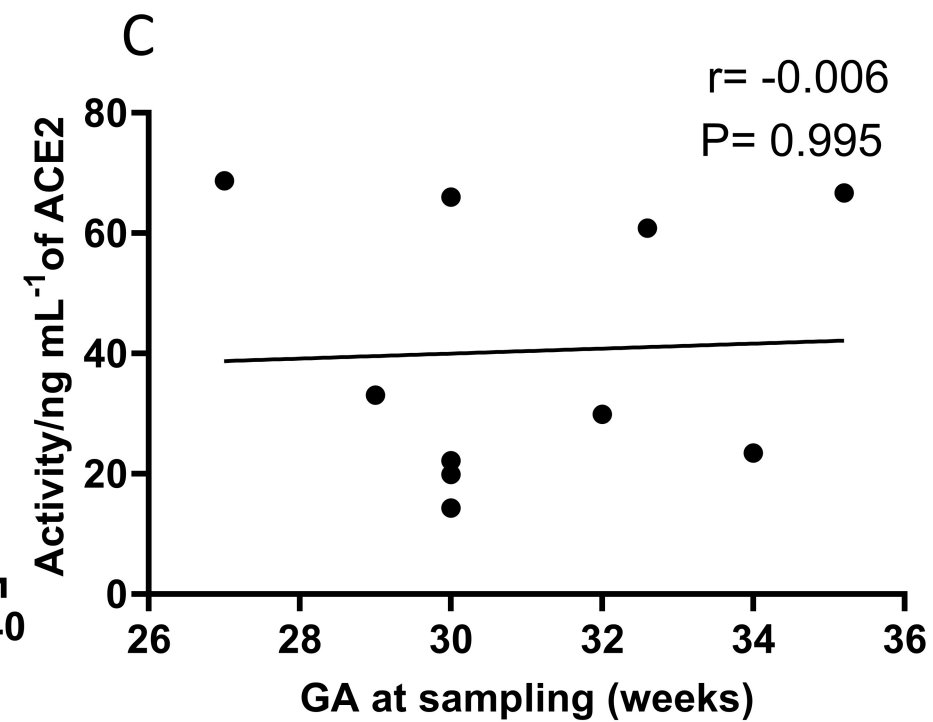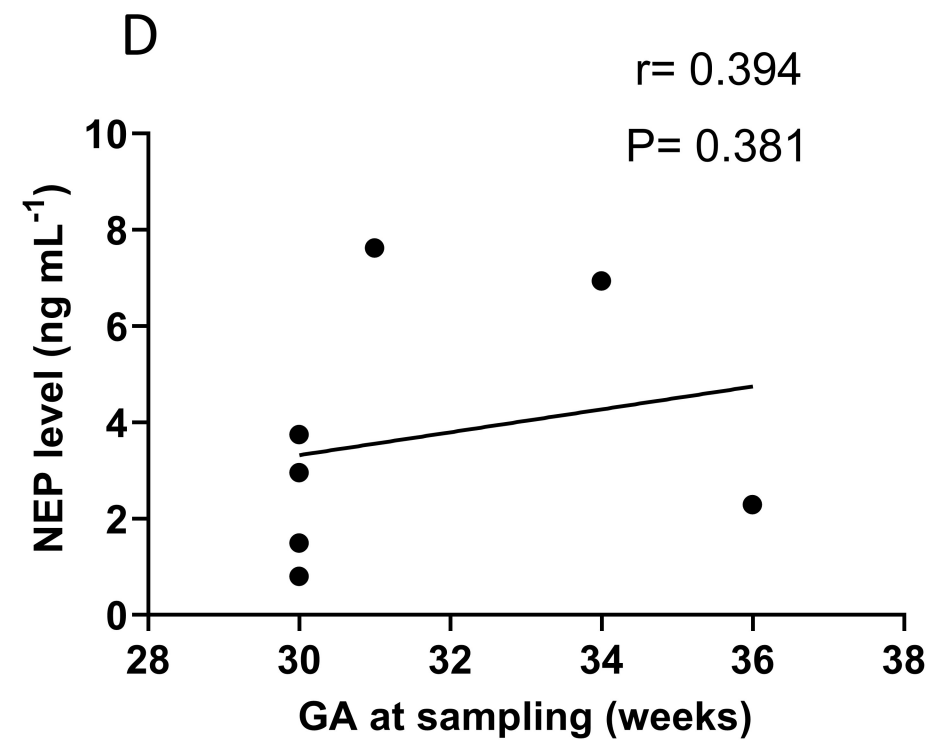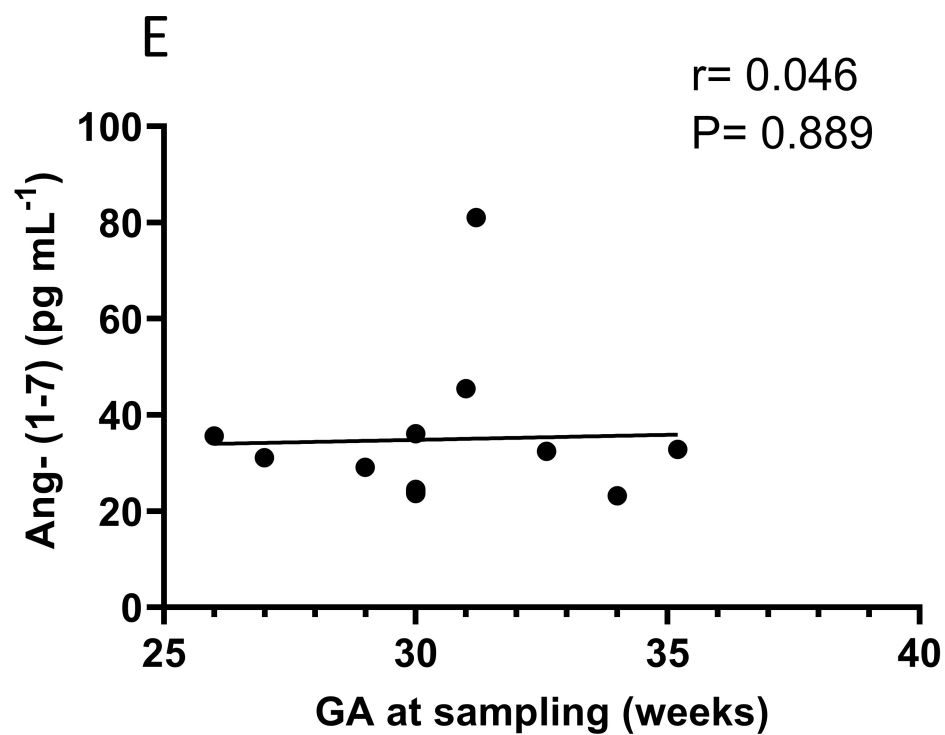

Supplement: Supplementary file 3 [file Data_Sheet_3.PDF]
